# Prevalence of Hypertension and Determination of Its Risk Factors in Rural Delhi

**DOI:** 10.1155/2016/7962595

**Published:** 2016-04-03

**Authors:** Jugal Kishore, Neeru Gupta, Charu Kohli, Neeta Kumar

**Affiliations:** ^1^Community Medicine, Vardhman Mahavir Medical College, New Delhi 110029, India; ^2^Indian Council of Medical Research, New Delhi 110029, India; ^3^Community Medicine, Maulana Azad Medical College, New Delhi 110002, India

## Abstract

*Introduction*. Hypertension is an important public health challenge in both economically developing and developed countries. It is one of the risk factors for cardiovascular mortality. Data is available on hypertension in urban population but few studies are reported in rural areas.* Materials and Methods*. It was a community based cross-sectional study conducted in two rural areas in Delhi among 1005 subjects selected using systematic random sampling method. WHO STEPS approach was used to collect data. Blood pressure, body mass index, and blood sugar were measured. Data analysis was done using SPSS version 16. Odds of hypertension among subjects with risk factors were calculated. *p* value less than 0.05 was considered significant.* Results*. The prevalence of hypertension was 14.1% among study subjects. Hypertension was significantly higher in individuals more than 35 years than those less than 35 years. Hypertension was significantly higher in those who take alcohol and in subjects with raised total cholesterol level but in multivariate analysis only age, education, and cholesterol levels were independently associated with hypertension.* Conclusion*. There is significant burden of hypertension in rural areas in Delhi. Age, education, and cholesterol levels were independent risk factors of hypertension.

## 1. Introduction

Hypertension (HTN) is an important public health problem in both economically developed and developing nations [1]. As per World Health Organization report, about 40% of people aged more than 25 years had hypertension in 2008 [2]. Danaei et al. studied secular trends in the age-adjusted mean systolic blood pressure (SBP) worldwide. Their findings revealed mean age-adjusted SBP declined by approximately 2 mmHg from 1980 to 2008. The age-adjusted SBP was highest in low-income and middle-income countries. In the same period, mean age-adjusted SBP declined in economically developed regions such as Australia, North America, and Western Europe and increased in economically developing regions such as Oceania, East Africa, and South and Southeast Asia. Additionally, due to the growth and aging of the population around the world, the number of people with uncontrolled hypertension was reported to have increased between 1980 and 2008 [3].

Worldwide, 7.6 million premature deaths (about 13.5% of the global total) were attributed to high blood pressure. About 54% of stroke and 47% of ischemic heart disease worldwide were attributable to high blood pressure [4]. Hypertension has been associated with increased risk of coronary artery disease and is an independent risk factor for cardiovascular and cerebrovascular diseases [5, 6]. A meta-analysis also reported that lower values of blood pressure are associated with higher risk of cardiovascular disease and also with chronic kidney diseases [7, 8]. The situation is critical in the Southeast Asian region with studies reporting HTN as an important risk factor for attributable burden of disease in the region [9, 10]. An alarming rise in HTN projected by the Global Burden of Hypertension 2005 study and the GBD 2010 study portrays a grim picture for the Indian population [2, 9]. Beyond programs aimed at the prevention of hypertension, treatment of hypertension remains a challenge in many parts of the world. Looking at the existing burden of disease, the Indian Government has launched the National Program for Prevention and Control of Cancer, Diabetes, Cardiovascular Diseases and Stroke for prevention and control of disease at community level [11].

Reliable information about the prevalence of hypertension is essential to the development of national and local level health policies for prevention and control of hypertension. Community level data for hypertension and its risk factors is scarce in Delhi. Thus, this study was conducted with the objective of finding prevalence of hypertension and its risk factors in a rural area in Delhi.

## 2. Materials and Methods

### 2.1. Study Design, Setting, and Sample Size

It was a community based cross-sectional study conducted in two rural field practice areas, covered under the Department of Community Medicine, Maulana Azad Medical College, New Delhi. Study population was constituted by all people above 18 years of age residing in two villages of Delhi.

### 2.2. Sample Size and Samplings

The sample size was calculated on the basis of a previous study which recorded prevalence of hypertension in rural population as 15% [12]. Taking 95% confidence interval, the required sample size for an 18,800 population was 194 (derived by Epi-Info software version 7). However, a total of 1005 subjects were included in the study. Systematic random sampling was used to select study subjects in the two villages.

### 2.3. Study Instruments and Data Collection

A predesigned, pretested, semistructured questionnaire was used by the surveyors containing items to assess sociodemographic profile like age, sex, identification data, socioeconomic status, and so forth. The WHO STEPS approach was employed to study the profile of the hypertension in the population. STEPS approach includes three sequential phases.

Information on sociodemographic variables and behavioural risk factors was collected, that is, tobacco use, alcohol use, and related factors using a questionnaire (STEP  1); clinical measurements such as weight, height, and blood pressure were obtained using standardized protocols and instruments (STEP  2); cholesterol, triglycerides, and high density lipoprotein (HDL) were measured (STEP  3) [13]. The standard WHO STEPS questionnaire was pretested before the study. It was adapted by including local terms and translated into local (Hindi) language and back translated into English by Hindi and English expert and field tested on 20 subjects in rural community. Self-reported history of use of tobacco as beedi or cigarette or any other form of tobacco and alcohol consumption as well as history of hypertension and diabetes mellitus was obtained from the respondents.

Blood pressure was recorded three times in sitting position, in the right arm, using a standard android dial BP apparatus (mercury type of BP apparatus is phased out from health care setting). The standard protocol was followed and the average of the last two readings was used in the analyses. Hypertensive subjects were defined as those with systolic blood pressure (SBP) equal to or more than 140 mmHg and/or diastolic blood pressure (DBP) equal to or more than 90 mmHg or those being treated for hypertension [13, 14].

Body mass index (BMI) was calculated as weight in kilograms divided by height in meters squared. Overweight and obesity were defined as BMI ≥ 23–24.9 kg/m^2^ and BMI ≥ 25 kg/m^2^, respectively [15]. Blood sugar fasting and postprandial and cholesterol levels were measured among study subjects.

### 2.4. Ethical Issues

Each selected subject was given explanation about the procedure and objectives of the study. Written informed consent was obtained and referral services were provided if required at the rural health centre. The prior ethical clearance for the study was obtained from the institutional ethics committee.

### 2.5. Statistical Analysis

Data analysis was done using SPSS version 16. The results were explained in simple proportions. Difference between groups was assessed using chi square test for their statistical significance. Odds were calculated in logistic regression analysis. *p* value less than 0.05 was considered significant.

## 3. Results

The prevalence of hypertension was 14.1% (142/1005) among study subjects.  Table 1 shows the sociodemographic characteristics such as age, sex, and religion of nonhypertensive (*n* = 863) and hypertensive (*n* = 142) groups. The hypertensive group was significantly higher in individuals more than 35 years than those less than 35 years. There was significant difference in the two groups with respect to age. There were predominantly Hindus in the study. The mean number of years of education for nonhypertensive cases was 8.6 years and for hypertensive cases it was 6.2 years. There was significant difference in hypertension prevalence in different education classes. There was no significant difference in the two groups in monthly per capita income but significant difference was seen in occupation categories.

Out of these 142 hypertensive subjects, previous history of hypertension was given by 68 (6.8%) of subjects and 50 (5.0%) subjects reported history of high BP records in the past one year. Out of these 68 subjects, only 29 (42.6%) reported that they were taking antihypertensive medications, 49 (72.1%) reported lesser intake of salt in diet, 25 (36.8%) were doing exercise for control of BP, and 43 (63.2%) were taking efforts for weight control for BP. Yield of this study was detecting 74 new cases of hypertension in the rural population of Delhi.

Table 2 shows risk factors among hypertensive and nonhypertensive groups. It can be seen that there was no significant difference in tobacco intake, both present and past tobacco use in the two groups. The hypertensive group was significantly higher in those who take alcohol than in the other group when inquiring about the alcohol intake in the past one year (*p* value = 0.02). Cholesterol levels were measured among study subjects. Hypertension was found in 20.7% of subjects with raised total cholesterol level and 11.1% among those with normal values which was statistically significant (*χ* ^2^ = 16.2, *p* value = 0.01). Similarly there was significant difference in raised triglyceride levels in the two groups. A significantly higher number of study subjects were hypertensive overweight and obese group as compared to the other group (*χ* ^2^ = 8.87, *p* value = 0.03). 12 (8.4%) were diabetic among hypertensive cases and 130 (91.5%) were nondiabetic.

Table 3 shows results of multivariate analysis for hypertension and its risk factors which showed that age, education, and cholesterol levels were independently associated with hypertension.

Multivariate analysis using logistic regression was used to find independent association of various factors. All the variables with *p* value less than or equal to 0.1 in univariate analysis were analysed in multivariate analysis. As shown in Table 3, age group less than 35 years has lesser odds of having hypertension than age group more than 35 years. In education classes, taking illiterate as baseline, those educated up to primary and high school level had significantly lesser odds of hypertension. Similarly, in occupation categories, taking unemployed as baseline, those who were retired had significantly higher odds of hypertension. Subjects with normal cholesterol levels had lesser odds of having hypertension than those with raised levels.

## 4. Discussion

The present study showed that the prevalence of hypertension was significantly higher in individuals more than 35 years as compared to those less than 35 years. Hypertension increase with the increase of age is a well-known fact now. Vasan et al. in their study conducted among 1298 subjects found significant association of hypertension with age [16]. There was significant difference in prevalence of hypertension in different education classes. Wang et al. also found that both systolic and diastolic blood pressure were inversely associated with the level of school education independent of all other risk factors [17]. Education makes the people aware of the disease and what precautions can be undertaken by the healthy individual. No significant association was found between hypertension and monthly per capita income but significant differences were found in different occupation classes. These are consistent with the findings in study conducted by Tsutsumi et al. which revealed that occupation and related stress were as independent risk factor of hypertension [18]. Univariate analysis showed hypertension more prevalent in semiprofessional and professional classes. Retired personnel had higher proportions of hypertension due to age. The confounding factors were adjusted in multivariate analysis which showed that only retired subjects had significantly higher odds of hypertension. The present study showed that only 42.6% of subjects who were known hypertensives were taking antihypertensive medications. Similar results were stated by Kusuma et al. in their study carried out in Delhi and found that only 59% of hypertension patients were on medication [19]. This difference could be due to our study population that belongs to a rural area where tendency of not starting long term medication is always present. Among modifiable factors, no significant association was shown with tobacco intake. This is inconsistent with findings of other studies where tobacco use has been found to be associated with hypertension [20, 21]. This could be due to the low level of tobacco use in this population as compared to national average of 35% which is around 48% in males and 20% in females while in the present study it is only approximately 11% [11]. Hypertension was significantly higher in individuals who take alcohol than those who did not. Alcohol has been reported as an independent risk factor by other authors as well [22, 23]. Similarly hypertension was more prevalent among those with raised cholesterol and triglycerides levels. Similar results were shown by other studies also previously [23]. The prevalence of hypertension was found to be consistently increased with increasing BMI as revealed by other authors [16, 24]. In multivariate analysis, raised total cholesterol, age more than 35 years, and education were independent risk factors of hypertension as reported by other authors as well [25].

## 5. Limitations

Lack of specific data on stress levels is a major limitation of the study. Any causal association cannot be derived from the present cross-sectional study design. More research with appropriate study design is needed to find if any causal association exists between hypertension and the discussed variables.

## 6. Conclusion and Recommendation

It can be concluded that there is significant burden of hypertension in rural areas in Delhi. Age, education, and cholesterol levels were independent risk factors of hypertension in the present study. Education level of people should be raised to reduce the prevalence. Cholesterol levels should be cut down using approaches of behaviour change communication (BCC) in community. Immediate measures should be taken for prevention and control of hypertension in Delhi.

## Figures and Tables

**Table 1:** Sociodemographic characteristics of the study subjects.

Variable	Subgroups	Group				*χ* ^2^, *p* value
		Nonhypertensive		Hypertensive		
		*n* = 863	%	*n* = 142	%	
Gender	Male	337	86.2	54	13.8	0.05, 0.8
	Female	526	85.7	88	14.3	

Age	Less than 35 years	428	95.3	21	4.7	59.4, 0.01
	More than 35 years	435	78.2	121	21.8	

Religion	Hindu	842	85.9	138	14.1	6.13, 0.04
	Others	21	87.5	4	12.5	

Education level	Primary	20	95.2	1	4.8	36.9, 0.01
	Middle	179	87.3	26	12.7	
	High school	217	87.9	30	12.1	
	Junior college	151	90.4	16	9.6	
	Graduate	103	90.4	11	9.6	
	Postgraduate	38	95	2	5	
	Illiterate	155	73.5	56	26.5	

Monthly per capita income	Up to Rs. 1000	322	87.7	45	12.3	2.5, 0.47
	Between Rs. 1001 and Rs. 2000	227	86.3	36	13.7	
	Between Rs. 2001 and Rs. 5000	244	83.8	47	16.2	
	More than Rs. 5001	70	83.3	14	16.7	

Occupation	Professional	57	86.4	9	13.6	22.4, 0.01
	Semiprofessional	15	78.9	4	21.1	
	Clerical, shop-owners, farm owners	23	95.8	1	4.2	
	Skilled worker	33	91.7	3	8.3	
	Semiskilled worker	58	90.6	6	9.4	
	Unskilled worker	123	84.2	23	15.8	
	Housewife	415	85	73	15	
	Retired	9	52.9	8	47.1	
	Unemployed	130	89.7	15	10.3	

**Table 2:** Modifiable risk factors of hypertension in study subjects.

Variable	Subgroups	Group				*χ* ^2^, *p* value
		Nonhypertensive		Hypertensive		
		*n* = 863	%	*n* = 142	%	
Present tobacco use	Yes	92	82.1	20	17.9	1.4, 0.2
	No	771	86.3	122	13.7	

Past tobacco use	Yes	3	75	1	25	0.4, 0.5
	No	860	85.9	141	14.1	

Alcohol use ever	Yes	48	78.7	13	21.3	2.9, 0.2
	No	815	86.4	129	13.6	

Alcohol use in the past one year	Yes	35	72.9	13	27.1	6.9, **0.02**
	No	828	86.5	129	13.5	

Total cholesterol	Raised	249	79.3	65	20.7	16.2, **0.01**
	Normal	614	88.9	77	11.1	

HDL	Decreased	824	85.7	138	14.3	0.86, 0.35
	Normal	39	90.7	4	9.3	

Triglycerides	Raised	177	80.5	43	19.5	6.8, **0.01**
	Normal	680	87.4	98	12.6	

Body mass index	Underweight	95	91.3	9	8.7	8.87, **0.03**
	Normal	272	89.2	33	10.8	
	Overweight	133	83.6	26	16.4	
	Obese	363	83.1	74	16.9	

Brisk walk or cycling daily for 30 minutes	Yes	700	86.5	109	13.5	1.47, 0.2
	No	163	83.2	33	16.8	

**Table 3:** Multivariate analysis for risk factors of hypertension.

Variable	Subgroups	Odds ratio (95% confidence interval)	*p* value
Age	Less than 35 years	Reference	0.01
	More than 35 years	3.60 (2.11–6.15)	

Religion	Hindu	Reference	
	Others	0.89 (0.17–2.73)	0.60

Education level	Illiterate	Reference	
	Primary	0.14 (0.01–1.12)	0.06
	Middle	0.43 (0.25–0.75)	0.01
	High school	0.35 (0.19–0.63)	0.01
	Junior college	0.23 (0.11–0.47)	0.01
	Graduate	0.25 (0.10–0.63)	0.01
	Postgraduate	0.07 (0.01–0.40)	0.01

Occupation	Unemployed	Reference	
	Retired	4.00 (1.21–13.15)	0.02
	Housewife	0.72 (0.36–1.43)	0.35
	Unskilled worker	1.37 (0.63–2.97)	0.41
	Semiskilled worker	0.53 (0.17–1.68)	0.28
	Skilled worker	0.52 (0.13–2.07)	0.35
	Clerical, shop-owners, farm owners	0.29 (0.03–2.74)	0.28
	Semiprofessional	1.35 (0.35–0.24)	0.65
	Professional	2.54 (0.88–7.34)	0.08

Alcohol use in the past one year	Yes	Reference	
	No	0.81 (0.34–1.54)	0.34

Total cholesterol	Normal	Reference	
	Raised	1.64 (1.12–2.45)	0.03

Triglycerides	Normal	Reference	
	Raised	0.91 (0.57–1.48)	0.68

Body mass index	Underweight	Reference	
	Normal	1.31 (0.62–3.12)	0.48
	Overweight	1.98 (0.89–4.12)	0.18
	Obese	1.61 (0.78–3.71)	0.26

Diabetes mellitus	No	Reference	
	Yes	2.01 (0.96–4.16)	0.06
